# Serum Neopterin Is Not Influenced by Age and Blood Group of Transfusion-Transmitted Infection Negative Blood Donors

**DOI:** 10.7759/cureus.9636

**Published:** 2020-08-10

**Authors:** Ahsan Ashfaq, Ayesha Ejaz, Fareena Khalil Ahmed, Noor Un Nisa, Suresh Langhani, Sumaira Riffat, Madiha Ariff

**Affiliations:** 1 Physiology, Liaquat National Medical College, Karachi, PAK; 2 Nephrology, Jinnah Postgraduate Medical Centre, Karachi, PAK; 3 Physiology, Sir Syed College of Medical and Dental Sciences for Girls, Karachi, PAK; 4 Physiology, Jinnah Sindh Medical University, Karachi, PAK; 5 Pathology, Jinnah Sindh Medical University, Karachi, PAK; 6 Internal Medicine, Dow University of Health Sciences, Karachi, PAK

**Keywords:** transfusion transmitted infections, screening, neopterin

## Abstract

Objectives

To measure serum neopterin levels in blood donors of the local population and to study its relationship with age and blood group of transfusion-transmitted infection (TTI) negative blood donors.

Methodology

This cross-sectional study was carried out in the Department of Physiology at Liaquat National Hospital and Medical College (LNMC), Karachi, Pakistan, in collaboration with the Basic Medical Sciences Institute and Jinnah Post Graduate Medical Centre (JPMC). Data were collected from January 2018 to July 2018. A total of 174 blood donors participated in the study, who were selected by using a random sampling technique. They were screened through the standard procedures used for screening at the JPMC blood bank for TTIs as per the World Health Organization recommendations. Serum neopterin was estimated using enzyme-linked immunosorbent assay (ELISA) kits. Data analysis was performed using Statistical Package for Social Sciences (SPSS) version 23 (IBM Corp., Armonk, NY, USA). Chi-square and ANOVA (analysis of variance) were applied, and tests of significance were kept as P < 0.05.

Results

Neopterin level in the serum of TTI-positive blood donors was 15.1 ± 4.59 nmol/L, which was above the normal range and cutoff value of 10 nmol/L, whereas the neopterin level in the serum of TTI-negative blood donors was 6.1 ± 1.82 nmol/L (P = 0.001). In TTI-negative blood donors, the neopterin levels were within normal limits and were not influenced by age and blood groups (P > 0.05).

Conclusions

Serum neopterin levels did not report any significant difference in terms of age and blood group of TTI-negative blood donors and were seen to be within normal limits.

## Introduction

Transfusion of blood is a vital element of health care that can save millions and millions of lives; nonetheless, this life-saving technique might seldom be coupled with a risk of transmitting disease from the infected donor to the recipient [1]. Therefore, with each blood transfusion, there exists a potential risk for acquiring a transmissible disease. Blood-borne transfusion infections are most importantly associated with the transfusion-associated disease, specifically in developing countries. Knowing the pathology of infection-causing pathogens, especially the infections that are endemic in a population, is important to minimize the risk of transmission of such infections [2]. It is important to screen for transfusion-transmitted infections (TTIs) for ensuring a safe transfusion of blood. Speaking from a human and economical perspective, unsafe transfusion of blood is damaging for both [3]. With transfusion of each unit of blood, there exists a 1% chance of transfusion-related complications, which included TTIs such as hepatitis B virus (HBV), hepatitis C virus (HCV), human immune-deficiency virus (HIV), malaria, and syphilis [4]. In countries such as Pakistan, where the presence of voluntary blood donors is limited, much dependency is laid on replacing and paying donors, in addition to a lack of systematic screening strategies [5]. Furthermore, the high prevalence of HBV, HCV, and malaria adds more problems to transfusion-related issues. In comparison to voluntary donors, commercially acquired blood donors carry more chances of infection and therefore more prone to transmit TTIs. Far-reaching consequences are observed in both recipients and their families with regard to the transfusion of infected blood-related morbidity and mortality [6].

Concerning local blood donors, researchers have reported much higher incidences of blood transfusion infections from commercially acquired blood donors. Approximately 20% are found to have HCV and around 10% HBV infection [7]. Among donors from family replacements, the incidence of HBV is estimated at 5% and that for HCV infection is 2.5%, whereas in voluntary blood donors, reported prevalence was 2% for HBV and 0.5% for HCV [8]. To screen blood that is donated, the World Health Organization (WHO) recommends core testing of hepatitis B surface antigen (HBsAg), hepatitis C antibody, serology for syphilis, and HIV subtypes 1 and 2. Nonetheless, specific tests cannot be performed on every blood donor for newly emerging infections or unrecognized infections [9]. A potentially hazardous pathogen might stay undetected either due to their known pathology but not being screened or because they aren’t known yet; therefore, the present screening technology tends to be non-effective against such pathogens. It might also be a possibility that the donated blood is in the window period, where the donor is even though diseased but the production of specific antibody is not yet started to be detected in the blood. Non-specific screening through immune markers such as neopterin might help in reducing this threat [10].

Neopterin is regarded as a sensitive indicator for activated cell-mediated (T-helper cell type-1) immune response. Immune system activation with subsequent increase in levels of neopterin is the key feature of multiple pathological states, especially viral infections. Because neopterin remains stable in biological fluids, its use for the evaluation of cell-mediated immune response intensity can be made to good effect [2]. Ample studies throughout the globe have established neopterin’s role as a biomarker of onset, progression, and outcome of various diseases. For instance, rising serum neopterin levels are reported in patients with chronic HBV infection. Among hepatitis B carriers, elevated levels of neopterin might indicate replication. Among patients with HCV infection, raised neopterin levels are usually linked to a positive polymerase chain reaction (PCR) [11]. Serum levels of neopterin are seen elevated among HIV patients both in the acute phase as well as in the window period, correlating both extent and severity of the disease. Researches have shown that high serum neopterin levels are also reported in dengue fever, which carries a potential transmission risk through transfusion. Even in malaria, elevations in serum neopterin levels have been reported [12].

With multiple trials over the years, nationwide screening of neopterin level elevation was made mandatory in Austria in 1994, where the significant role of neopterin in the screening of blood was recognized. After that, many researchers from not only Austria but also other countries have demonstrated the capabilities of screening for neopterin levels for improving the safety of blood donations with regard to viral infection transmission [13]. Research data suggest that neopterin concentrations in blood donors differ between AB0 blood group phenotypes [14], but no such data are available for our population. Since we are advocating the use of additional neopterin screening as a safety marker in blood donations in this part of the world, we must first explore whether neopterin levels significantly changes with the blood group of the blood donor or not. By exploring this fact and finding that neopterin is not influenced significantly with ABO blood groups in our population and does not reach the cutoff value of 10 nmol/L due to blood group phenotype, we are demonstrating that no false-positive elevation beyond the cutoff value is associated with variation in blood groups of the blood donors. Hence, only the TTIs raise neopterin beyond the cutoff value in the blood donor population. The objective of this study was to estimate that serum neopterin levels do not correlate with age or blood group of TTI negative blood donors.

## Materials and methods

After receiving approval from the ethical review committee, a cross-sectional prospective study was carried out at the Physiology Department of Liaquat National Hospital And Medical College (LNMC), Karachi, Pakistan, in collaboration with the Basic Medical Sciences Institute and blood bank of Jinnah Postgraduate Medical Center (JPMC). The duration of the study was six months, in which blood donors between 18 and 60 years of age who consented for participating in the study were included and enrolled through a random sampling method. After the routine screening of blood donors using standardized procedures was conducted at the blood bank of JPMC, which are as per the WHO guidelines, 5 mL of blood was collected and centrifuged with serum collected and stored at -20ºC to determine the level of serum neopterin. Using the enzyme-linked immunosorbent assay (ELISA) method, the levels of serum neopterin were calculated. The cutoff value for serum neopterin was <10 nmol/L, which was taken as normal for healthy blood donors. Besides anthropometric data, vitals and demographics were also recorded.

For data analysis, Statistical Package for Social Sciences (SPSS) Version 23 (IBM Corp., Armonk, NY, USA) was used. Clinical characteristics were summarized in terms of frequencies and percentages for qualitative/categorical variables and mean ± SD for quantitative variables. A chi-square test was used to determine the association between qualitative and categorical variables, and Student’s t-test/ANOVA was used for quantitative data. A P-value of <0.05 was considered significant.

## Results

Out of a total of 174 patients, 151 (86.8%) had a negative TTI screening test recommended by the WHO for routine screening tests, whereas in 23 (13.2%) of patients, these screening tests were positive and they had TTI. Among the negative screening test of blood donors, elevated levels of serum neopterin, i.e., >10 nmol/L, was reported in 1 (0.7%) patients, whereas in positive screening test of blood donors, elevations of neopterin were observed in 20 (86.9%) of patients. The mean serum neopterin level in negative screening test of blood donors was 6.1 ± 1.82 nmol/L and that in the positive screening test of blood donors was 15.1 ± 4.59 nmol/L. The neopterin level in TTI-positive donors was highly significant when compared to the neopterin level in healthy blood donors (P = 0.001) (Table 1).

**Table 1:** Neopterin levels in donors with positive and negative screening tests (n = 174) *Neopterin level in TTI-positive donors was highly significant when compared to the neopterin level in healthy blood donors (P = 0.001). TTI, transfusion-transmitted infection

Screening test	No. (%)	No. of positive with elevated neopterin level (>10 nmol/L)	Neopterin level (nmol/L)	
	n (%)	n (%)	Mean ± SD	P-value
Negative	151 (86.8)	1 (0.7%)	6.1 ± 1.82	0.001*
Positive	23 (13.2)	20 (86.9%)	15.1 ± 4.59	

**Figure 1:**
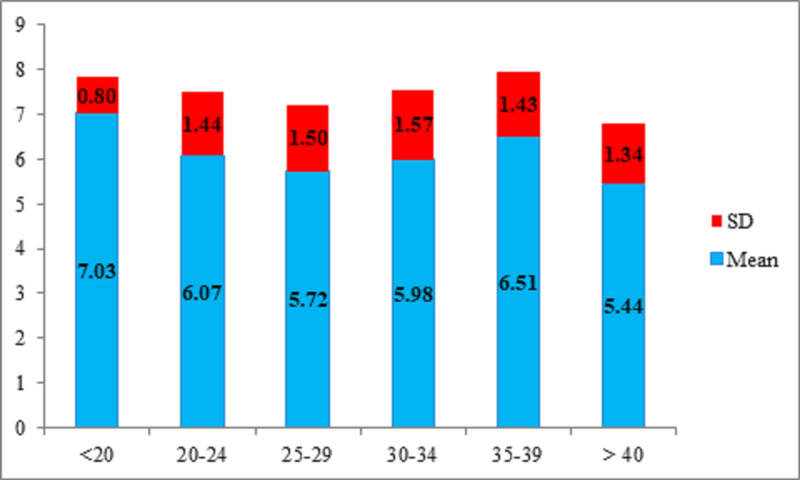
Neopterin levels in different age groups (normal) (n = 153)

Among the 153 patients with normal serum neopterin levels, 4 (2.6%) were below the age of 20 years, having mean serum neopterin levels of 7.03 ± 0.80 nmol/L, 50 (32.7%) were between 20 and 24 years of age, having mean serum neopterin levels of 6.07 ± 1.44 nmol/L, 52 (34%) were between 25 and 29 years of age, having mean serum neopterin levels of 5.72 ± 1.50 nmol/L, 34 (22.2%) were between 30 and 34 years of age, having mean serum neopterin levels of 5.98 ± 1.57 nmol/L, 10 (6.5%) were between 35 and 39 years of age, having mean serum neopterin levels of 6.51 ± 1.43 nmol/L, and 3 (2%) were above 40 years of age, having mean serum neopterin levels of 5.44 ± 1.34 nmol/L. The overall mean serum neopterin levels were 5.97 ± 1.48 nmol/L. Average neopterin levels according to age did not report the significant difference, i.e., P > 0.05 (Table 2, Figure 1).

**Table 2:** Neopterin levels in different age groups (normal) (n = 153) Average neopterin level (mean ± SD) according to age had no significant difference (P > 0.05). *n = number **% = percentage (frequency)

Variable	Neopterin level		
Age group (in years)	n*	%**	Mean ± SD
<20	4	2.6	7.03 ± 0.80
20-24	50	32.7	6.07 ± 1.44
25-29	52	34.0	5.72 ± 1.50
30-34	34	22.2	5.98 ± 1.57
35-39	10	6.5	6.51 ± 1.43
>40	3	2.0	5.44 ± 1.34
Total	153	100.0	5.97 ± 1.48

**Figure 2:**
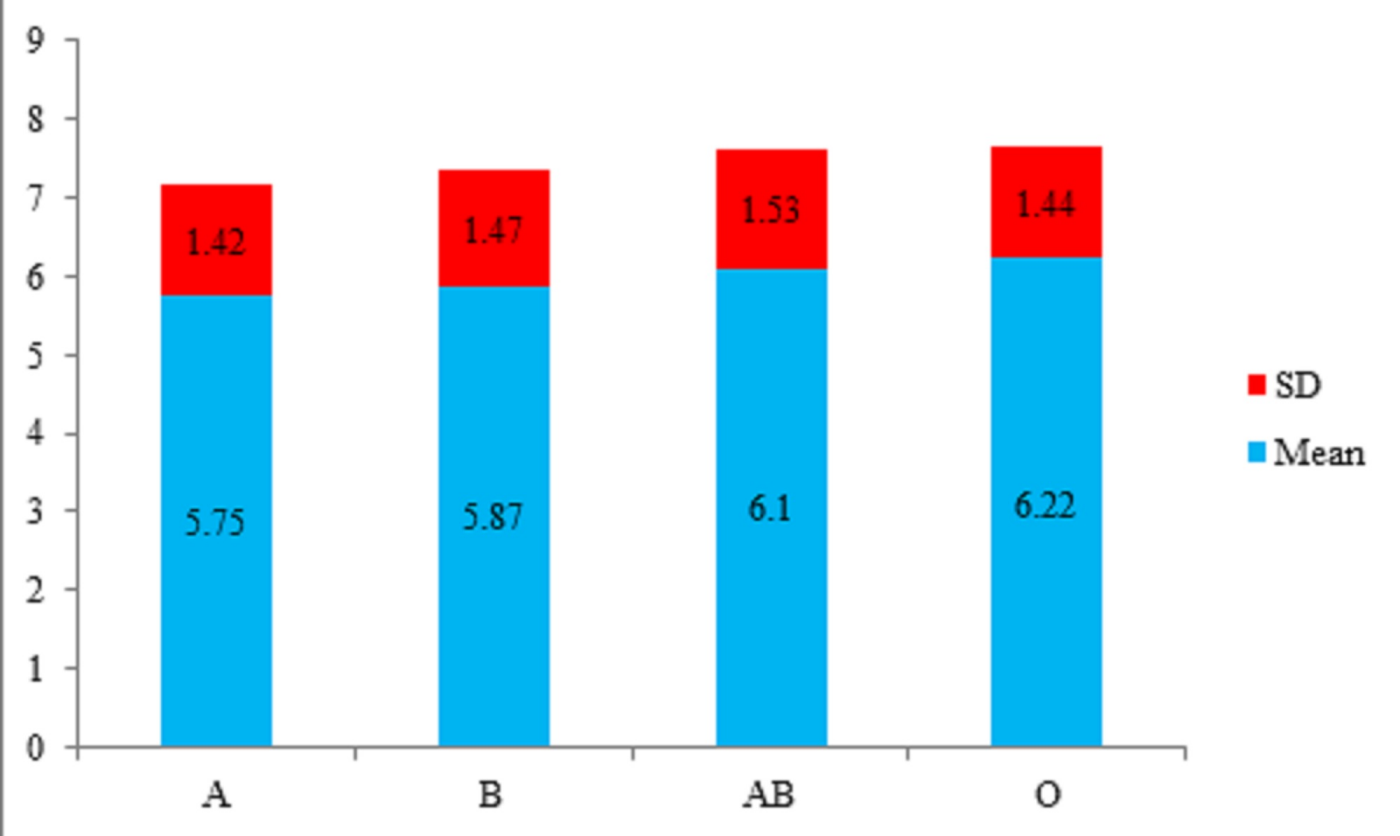
Neopterin levels in the subpopulation of different blood groups (normal) (n = 153)

With regard to normal serum neopterin levels in the subpopulation of different blood groups, from 153 blood donors, 40 (26.1%) reported blood group A with mean neopterin levels of 5.75 ± 1.52 nmol/L, 49 (32%) had blood group B with mean neopterin levels of 5.87 ± 1.47 nmol/L, 16 (10.5%) reported blood group AB with mean neopterin levels of 6.10 ± 1.53 nmol/L, and 48 (31.4%) reported blood group O with mean neopterin levels of 6.22 ± 1.44 nmol/L (Table 3, Figure 2).

**Table 3:** Neopterin levels in the subpopulation of different blood groups (normal) (n = 153) Average neopterin level (mean ± SD) according to blood group had no significant difference (P > 0.05). *n = number **% = percentage (frequency)

Variable	Neopterin level		
Blood group	n*	%**	Mean ± S.D
A	40	26.1	5.75 ± 1.52
B	49	32.0	5.87 ± 1.47
AB	16	10.5	6.10 ± 1.53
O	48	31.4	6.22 ± 1.44
Total	153	100.0	5.97 ± 1.48

## Discussion

According to the results of our study, out of 174 study participants, 13.2% reported elevated levels of serum neopterin. Other studies have reported incidences of elevated serum neopterin levels ranging from 1.6% to around 19.1% [15]. Likewise, a study reported that elevated serum neopterin levels significantly correlated with blood donors having HCV infection in addition to aspartate aminotransferase (AST) and alanine aminotransferase (ALT) levels of liver profile. Similarly, significant correlations of neopterin levels were reported with HIV infection as well [16]. High levels of neopterin were also found linked with other diseases such as viral infection and rejection. In another study by Zoller et al., mean serum neopterin levels were 7.8 ± 8.7 nmol/L, which were significantly higher than that in the control group blood donors [17].

A study determined the correlation between age, ALT, and AST in terms of serum neopterin levels between the healthy control group and HCV positive group and reported a significant difference of neopterin levels in terms of ALT and AST but did not report any significant difference between serum neopterin levels in terms of age groups [18]. Likewise, Parrak et al. observed an insignificant difference between serum neopterin levels with age or blood group of donors [19]. Similarly, in our study as well, serum neopterin levels were significantly higher in HCV infection donors than healthy donors but did not report the significant difference of serum neopterin levels in terms of age and blood group of donors.

In the blood donors of this study, three (1.7%) donors tested positive for cytomegalovirus (CMV) immunoglobulin M (IgM). In contrast, another study reported a higher frequency (3.7%) of CMV IgM positive blood donors having elevated neopterin levels [20]. In yet another study, 5.26% from 1,767 blood donations had an increase in levels of serum neopterin in CMV IgM positive patients, denoting an acute infection. However, in some studies, seroconversion was reported in around 10 cases having initial elevations in neopterin levels but later on reporting low neopterin levels, which might indicate neopterin elevations preceding the appearance of CMV antibodies by around two to four weeks [21]. Similarly, a study reported substantially higher levels of neopterin in early infection in comparison to late infection or even in the carrier state. All early infections, i.e., seroconversions, were found to have neopterin levels higher by 17% compared to late or carrier states. Recently, research reported elevated serum neopterin levels in 61% of CMV DNA positive cases. However, none of the studies reported any significance of neopterin levels to age or blood group [22].

Besides, other studies have also reported substantial differences in levels of neopterin among symptomatic dengue patients, much higher than those levels seen with measles or influenza virus infection. The levels also significantly correlated with the severity of disease and duration of fever but insignificant with age and blood group [23]. A study reported that out of 328 HIV-infected individuals, 64.7% had elevated levels of serum neopterin, i.e., >10 nmol/L [24]. In our study, HIV-positive donors also reported high levels of serum neopterin. In another study, where levels of serum neopterin were determined before and after induction of highly active antiretroviral therapy (HAART) for viral infections, a significant decline of neopterin levels with HAART therapy was reported. However, the patients who discontinued HAART reported again elevation in their serum neopterin levels. However, none of the studies reported any significance of neopterin levels to age or blood group [25].

Although the study determined the correlation of neopterin levels with age and blood group, the study was not immune from selection and observer bias, and due to the fact that the study was conducted with limited sample size, further studies with larger sample size would help in providing results with greater accuracy.

## Conclusions

In our study, serum neopterin level is significantly higher in the younger age group and in patients with O blood type. However, the association of the serum neopterin level with various age groups was insignificant. The relationship between serum neopterin level and the different blood groups has also been statistically insignificant. Therefore, blood screening can be used as an additional safety marker for non-specific screening of blood donations, as it is not influenced by the age and blood group of blood donors who are tested negative for transfusion-borne infections.
